# Protective Efficacy of Neutralizing Monoclonal Antibodies in a Nonhuman Primate Model of Ebola Hemorrhagic Fever

**DOI:** 10.1371/journal.pone.0036192

**Published:** 2012-04-27

**Authors:** Andrea Marzi, Reiko Yoshida, Hiroko Miyamoto, Mari Ishijima, Yasuhiko Suzuki, Megumi Higuchi, Yukie Matsuyama, Manabu Igarashi, Eri Nakayama, Makoto Kuroda, Masayuki Saijo, Friederike Feldmann, Douglas Brining, Heinz Feldmann, Ayato Takada

**Affiliations:** 1 Laboratory of Virology, Rocky Mountain Laboratories, Division of Intramural Research, National Institute of Allergy and Infectious Diseases, National Institutes of Health, Hamilton, Montana, United States of America; 2 Division of Global Epidemiology, Hokkaido University Research Center for Zoonosis Control, Sapporo, Japan; 3 Division of Bioinformatics, Hokkaido University Research Center for Zoonosis Control, Sapporo, Japan; 4 Department of Virology 1, National Institute of Infectious Diseases, Tokyo, Japan; 5 Office of Operations Management, Rocky Mountain Laboratories, Division of Intramural Research, National Institute of Allergy and Infectious Diseases, National Institutes of Health, Hamilton, Montana, United States of America; 6 Rocky Mountain Veterinary Branch, Rocky Mountain Laboratories, Division of Intramural Research, National Institute of Allergy and Infectious Diseases, National Institutes of Health, Hamilton, Montana, United States of America; Virginia Polytechnic Institute and State University, United States of America

## Abstract

Ebola virus (EBOV) is the causative agent of severe hemorrhagic fever in primates, with human case fatality rates up to 90%. Today, there is neither a licensed vaccine nor a treatment available for Ebola hemorrhagic fever (EHF). Single monoclonal antibodies (MAbs) specific for *Zaire ebolavirus* (ZEBOV) have been successfully used in passive immunization experiments in rodent models, but have failed to protect nonhuman primates from lethal disease. In this study, we used two clones of human-mouse chimeric MAbs (ch133 and ch226) with strong neutralizing activity against ZEBOV and evaluated their protective potential in a rhesus macaque model of EHF. Reduced viral loads and partial protection were observed in animals given MAbs ch133 and ch226 combined intravenously at 24 hours before and 24 and 72 hours after challenge. MAbs circulated in the blood of a surviving animal until virus-induced IgG responses were detected. In contrast, serum MAb concentrations decreased to undetectable levels at terminal stages of disease in animals that succumbed to infection, indicating substantial consumption of these antibodies due to virus replication. Accordingly, the rapid decrease of serum MAbs was clearly associated with increased viremia in non-survivors. Our results indicate that EBOV neutralizing antibodies, particularly in combination with other therapeutic strategies, might be beneficial in reducing viral loads and prolonging disease progression during EHF.

## Introduction

Ebola virus (EBOV) has a non-segmented, single strand negative-sense RNA genome and, together with Marburg virus, constitutes the family *Filoviridae* [1]. EBOV causes severe hemorrhagic fever in humans and nonhuman primates (NHPs) with the highest human case fatality rates among hemorrhagic fever viruses. Currently, there is neither an effective prophylaxis nor treatment available for Ebola hemorrhagic fever (EHF). While all Marburg virus isolates currently belong to a single virus species, multiple EBOV species have been described [1], [2]. *Zaire ebolavirus* (ZEBOV), first identified in 1976, is the most virulent species with case fatality rates in humans approaching 90% and almost 100% lethality in experimental macaque models [1], the current gold standard animal model among several established ZEBOV disease models [3].

The EBOV transmembrane glycoprotein (GP) is responsible for both receptor binding and fusion of the virus envelope with the host cell membrane [4], [5], and the only known target for neutralizing antibodies against this virus. The presence of EBOV-neutralizing antibodies was confirmed in the sera of convalescent patients and experimentally infected NHPs [6], [7]. The protective efficacy of passive immunization with hyperimmune sera or purified polyclonal antibodies was evaluated using rodent models and shown to be effective in mice and guinea pigs, whereas evidence of protective efficacy in primates, including humans, remains elusive [6], [7], [8]. In contrast, we have shown that certain GP-specific antibodies enhance filovirus infection *in vitro*, a mechanism called antibody-dependent enhancement (ADE), and that convalescent serum, hyperimmune serum, and serum from vaccinated animals contain a mixture of neutralizing, enhancing, and neutral antibodies [9]–[11]. Therefore, it seems possible that ADE may diminish the efficacy of neutralizing antibodies [10], [12] and thus polyclonal serum may not aid in passive immune therapy for EBOV. To reduce the potential risks and inherent disadvantages in using whole polyclonal serum for passive immune therapy against EHF, the use of well-defined and characterized monoclonal antibodies (MAbs) seems more promising and perhaps better justified. Additionally, MAb production is easier to scale up with keeping the quality consistent while preparation of polyclonal serum is not. This is an important factor for commercial production of emergency immunotherapeutics. Quantities of any particular polyclonal serum are finite and serum from different animals would have to be pooled for a large supply.

Multiple ZEBOV GP-specific MAbs, including neutralizing antibodies, have been generated in the past and several MAb epitopes have been identified [13]–[16]. In particular, the recombinant human MAb KZ52, which was generated using phage display libraries constructed from RNA derived from convalescent ZEBOV patients [13], was shown to be protective in rodent models [17]; however, this MAb failed to protect rhesus macaques from lethal ZEBOV challenge even when the animals were given a high dose of the MAb (50 mg/kg) twice (1 day before and 3 days after challenge) [18]. We have generated two mouse MAbs, ZGP133/16.3 and ZGP226/8.1, that seem to recognize unique epitopes in GP, compared to MAb KZ52 [15], [16]. Pre- and post-exposure treatment with each of the two MAbs in rodent disease models resulted in complete or partial protection and sterile immunity in several of the pre-exposure treated animals [15], [19].

In this study, we genetically modified these two MAbs to create human-mouse chimeric MAbs (ch133 and ch226) and evaluated their protective potential in the rhesus macaque model of lethal ZEBOV infection. Prophylactic treatment with MAbs ch133 and ch226 combined intravenously resulted in reduced viral loads and partial protection, indicating that antibody therapy might have beneficial effects in EHF.

## Results

### MAbs ch133 and ch226 neutralize ZEBOV *in vitro*

**Figure 1:**
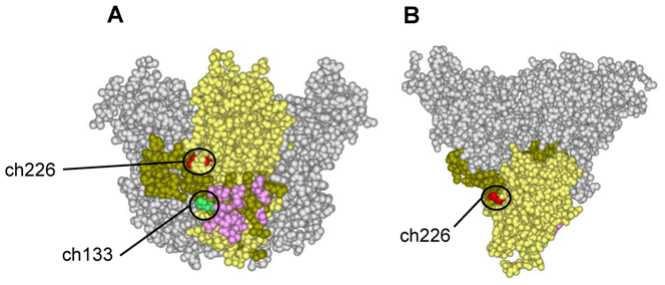
Locations of MAb epitopes. The trimeric structure of GP was constructed using the Discovery Studio 2.5 program (Accelrys, Inc.) based on the crystal structure of ZEBOV GP (PDB code: 3CSY). This structure lacks amino acid residues at positions 190–213, 311–312, 464–501, and 600–632, because no electron density was observed for these residues [16]. The molecular surfaces of the GP trimer are shown in side (A) and top (B) views. While one monomer is depicted as both subunits, GP1 (yellow) and GP2 (brown), the other two monomers are colored gray. The amino acid residues identified in escape mutants at positions 134 (ZGP226/8.1; ch226) and 549 (ZGP133/3.16; ch133) are shown in red and green, respectively. The epitope recognized by MAb KZ52 in the crystal structure of the GP-MAb complex is highlighted in pink.

In a previous study, we have identified different amino acid residues important for the neutralizing activity of the two mouse MAbs, ZGP133/16.3 and ZGP226/8.1, using a surrogate virus system [15]. All escape mutants selected in the presence of ZGP133/3.16 contained a single amino acid substitution at position 549 in the ZEBOV GP. In contrast, for ZGP226/8.1 three different escape mutants were isolated containing amino acid substitution at position 134, 194, or 199 in the ZEBOV GP, suggesting that this antibody recognizes a different conformational epitope. Mapping of these epitopes, together with that of KZ52, on the 3-D structure of the ZEBOV GP molecule indicates that these MAbs likely bind to different epitopes, although the ZGP133 epitope may partially overlap with that of KZ52 (Figure 1).

**Figure 2:**
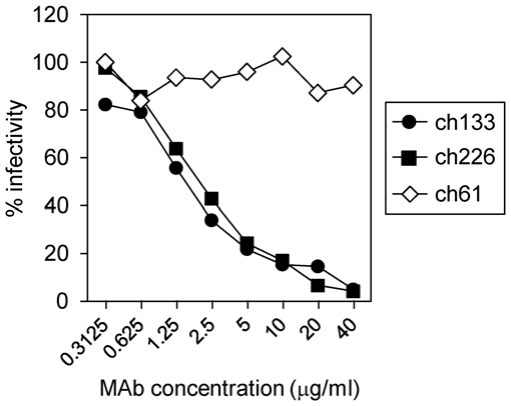
Chimeric MAbs neutralize ZEBOV. The indicated concentrations of MAbs were incubated with 200 focus-forming units of ZEBOV for 1 hour at 37°C and subsequently inoculated onto monolayers of Vero E6 cells in a 48-well plate. After incubation for 4 days at 37°C, cells were fixed and removed from BSL4 using standard operating procedures. Foci were stained with an anti-VP40 rabbit serum and a FITC-conjugated anti-rabbit IgG antibody, counted and titers were determined.

To evaluate the protective efficacy in nonhuman primates, we converted ZGP133/16.3 and ZGP226/8.1 into the human-mouse chimeric MAbs ch133 and ch226. MAb ch61 specific for the influenza virus hemagglutinin (HA) was generated as a control antibody. The neutralizing activities of chimeric MAbs ch133 and ch226 were analyzed *in vitro* by performing a focus reduction neutralization test [20]. Both MAbs significantly reduced the infectivity of ZEBOV in Vero E6 cells in a dose-dependent manner (Figure 2), whereas the negative control MAb (ch61) did not. The 50% inhibitory concentrations of ch133 and ch226 were 1.6 and 2.1 µg/ml, respectively. These values were similar to those of the original mouse MAbs (3.2 and 0.8 µg/ml, respectively) [19], indicating that genetic modification of these MAbs did not significantly affect their ability to neutralize ZEBOV *in vitro*.

### Protective efficacy in nonhuman primates

**Figure 3:**
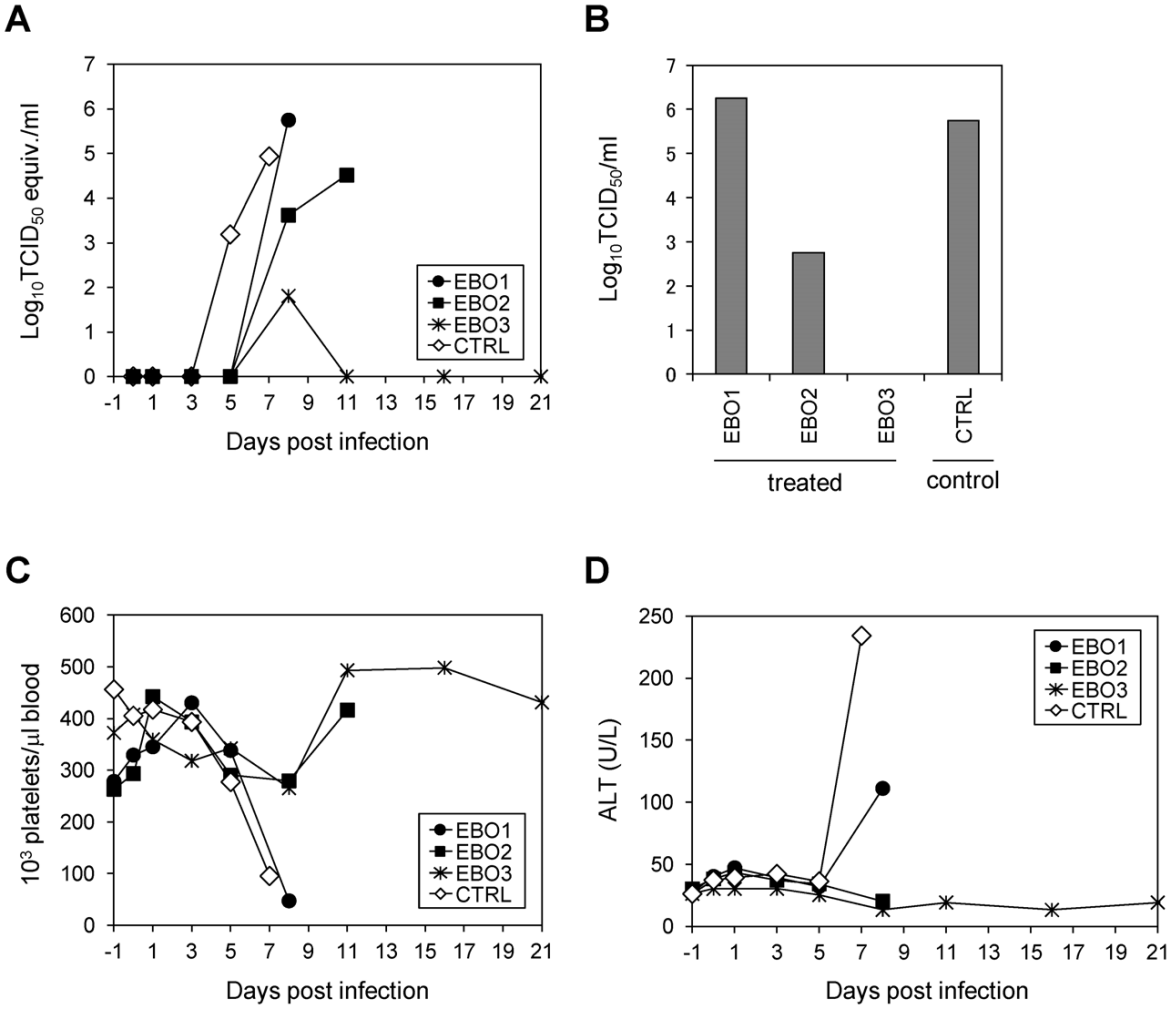
Hallmark laboratory parameters after ZEBOV challenge. Viral RNAs in the blood samples were detected as described in Materials and Methods (A). Virus titers in the blood samples collected 8 days after challenge were determined as TCID_50_ in Vero E6 cells (B). For CTRL, titers for the sample collected on day 7 are shown. Platelet counts (C) were determined from whole blood samples; alanine aminotransferase levels (ALT) (D) were determined from serum at the indicated time points.

The stability of the chimeric MAb was first tested *in vivo* by monitoring serum antibody levels in rhesus macaques that received 50 mg of the antibody intraveniously. The MAb half-life time in the serum was 3–4 days (data not shown). We next sought to evaluate the prophylactic efficacy of both MAbs combined in the well-established rhesus macaque model of EHF. Three rhesus macaques (EBO1, EBO2, and EBO3) were intraveniously treated with a mixture of MAbs ch133 and ch226 (25 mg of each MAb; 50 mg total) 24 hours before and 24 and 72 hours after challenge with a lethal dose of ZEBOV, strain Kikwit (10^3^ plaque-forming units). A control animal (CTRL) was identically challenged and treated at the same time points with MAb ch61 by the same route and dose. Animals CTRL and EBO1 developed fulminant EHF with viremia levels exceeding 10^4^ 50% tissue culture infectious dose (TCID_50_) equivalents/ml prior to day 8 and had to be euthanized on days 7 and 8, respectively (Figure 3A). This is a normal disease progression for rhesus macaques infected with a lethal dose of ZEBOV. Animal EBO2 showed a delayed onset of clinical signs and prolonged time to death with viremia levels still below 10^4^ TCID_50_/ml on day 8 (Figure 3B), although it had to be euthanized with characteristic signs of EHF on day 11. Furthermore, virus titers in liver, spleen, and adrenal gland were more than 1 log higher in the control animal (CTRL) compared to EBO2 (Table 1), again showing the delayed disease progression in this animal. Animal EBO3 was protected from clinical disease and survived. This animal had only very low level viremia detected by qRT-PCR on day 8 (Figure 3A); however, virus isolation was negative (Figure 3B). In addition, the survivor EBO3 showed no significant ZEBOV-specific changes in blood chemistry or hematology throughout the study; its liver enzyme levels (i.e. alanine aminotransferase (ALT)), as well as platelet counts, were always within the normal range (Figures 3C and 3D). To exclude viral escape under neutralizing pressure, we sequenced viral RNA isolated from blood collected on days 8 (EBO1) and 11 (EBO2). No mutation was found in the GP genes, indicating that virus escape did not occur as previously described for both MAbs *in vitro* [15].

**Table 1:** Virus titers in tissue samples collected from euthanized animals during the prophylactic treatment study.

NHP ID	Treatment	Time to death [days]	Liver	Spleen	Adrenal gland
CTRL	ch61	7	8.53^a^	8.38	8.24
BBO1	ch133+ch226	8	8.20	8.35	7.69
EBO2	ch133+ch226	11	7.32	6.75	7.05
EBO3	ch133+ch226	Survived	ND^b^	ND	ND

a Virus titers presented as log_10_ TCID_50_/gram tissue.

b ND, not done.

### Serum antibody levels in treated nonhuman primates

**Figure 4:**
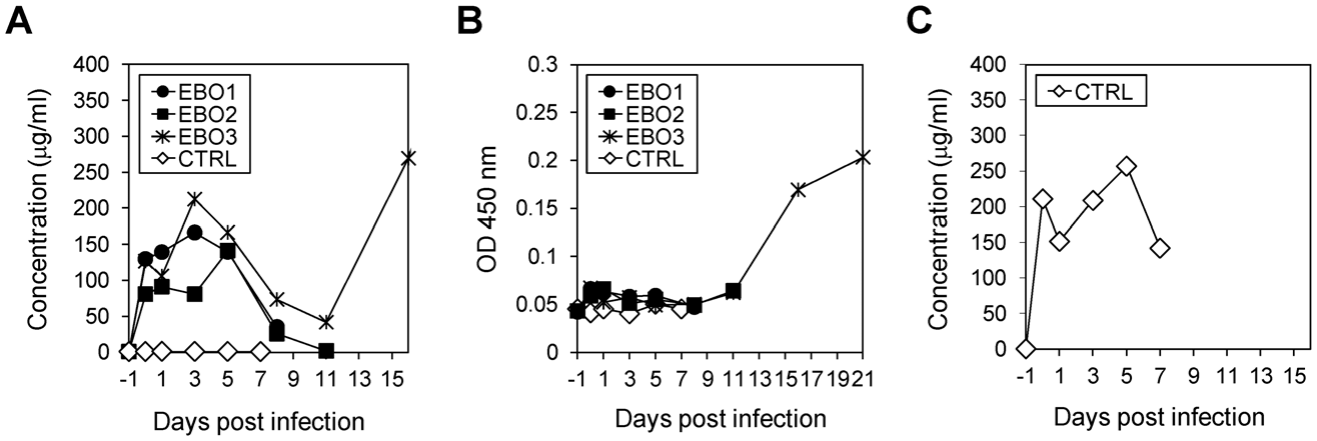
Serum antibody levels after ZEBOV challenge. Serum samples were collected from animals given the GP-specific MAb mixture or ch61 during the period of the experiments of prophylactic treatments. Antibodies specific to GP (A), NP (B), and influenza virus HA (C) were detected by ELISA, as described in Materials and Methods. Levels of anti-HA antibody were measured in the animal given only the control MAb (anti-HA, ch61). Spontaneous induction of GP-specific IgG in the surviving animal after day 11 was detected due to cross-reactivity of the secondary antibody used in the ELISA.

MAb concentrations in the serum samples collected throughout the experiment were monitored using enzyme-linked immunosorbent assay (ELISA). MAb concentrations (ch133 and ch226) on days 1–5 after challenge were maintained above 75 µg/ml, but drastically decreased to almost undetectable levels in animals EBO1 and EBO2 on day 8 (Figure 4A). The rapid decrease in serum MAb concentrations was timely associated with increased viremia in these non-survivors (Figures 3A and 4A). Notably, higher levels of MAb concentrations were detected in the serum of the surviving animal (EBO3), remaining above 50 µg/ml until day 11 before IgG concentrations steadily increased (Figure 4A). This increase is due to a challenge virus-induced IgG response against ZEBOV proteins leading to recovery from infection as demonstrated by the increase of nucleoprotein (NP)-specific IgGs (Figure 4B). The concentration of MAb ch61 in the serum of CTRL remained above 150 µg/ml until the animal had to be euthanized on day 7 indicating no consumption of neutralizing MAb through ZEBOV replication (Figure 4C).

## Discussion

Passive transfer of antibodies leads to either complete inhibition of virus replication, resulting in sterile immunity, or incomplete protection in which the virus replicates at a reduced level, allowing the host to mount virus-induced immune responses resulting in virus clearance. In this study, passive transfer of a mixture of two neutralizing MAbs conferred partial protection in rhesus macaques against lethal ZEBOV challenge with 1 of 3 animals surviving and another one showing delayed disease progression. Our data demonstrate that animals can be protected even when primary challenge virus replication occurs and that “sterile immunity" is not necessarily required for protection from lethal ZEBOV infection.

The GP gene of EBOV has two overlapping reading frames expressing the full-length transmembrane GP and two nonstructural soluble GPs (sGP and ssGP), which are secreted from ZEBOV-infected cells [21]–[24]. Due to its high abundance in the blood of infected humans, it has been proposed that sGP facilitates virus spread by deactivating anti-GP antibodies. Indeed, it has been shown that sGP can reduce the neutralizing activities of anti-GP serum *in vitro* [23]. A similar function was also suggested for the shed GP ectodomain resulting from GP cleavage on the cell or virus surface [25]. In this passive transfer study, serum MAb concentrations dropped remarkably at the terminal stage of the disease, indicating substantial consumption of these antibodies in the blood. Since both MAbs used in this study do not bind sGP dimers [15], this observation is likely due to uncontrolled virus replication leading to production of large quantities of the shed trimeric glycoprotein functioning as antibody decoy in the blood. Therefore, it seems that high levels of plasma antibodies are required to suppress virus replication until host immune responses are sufficiently induced. This could be achieved through additional injections (i.e. day 5 and day 7) or through higher MAb concentrations in the initial injections. Complete neutralization of ZEBOV using ch133 and ch226 *in vitro* was achieved at concentrations of greater than 40 µg/ml (Figure 2). The antibody dose used in this NHP study, which gave approximately 50–100 µg/ml blood at each treatment, did not significantly differ from the in vitro situation, but the half-life time of the MAb was less than 4 days. This again indicates that more than three injections (every 2–3 day) may have been beneficial. One possible reason for relatively short half-life of our chimeric MAbs might be reduced stability due to desialylation during the prolonged protein expression process in CHO cell culture. In general, sialic acids likely influence the solubility, thermal stability, and resistance to protease attack of various glycoproteins. Thus, it seems possible that the stability of MAbs may be improved by enhancing sialylation during expression in CHO cells [26].

It has been demonstrated that EBOV utilizes multiple cellular pathways for entry into host cells [27]. Direct inhibition of GP attachment to cell surface or endosomal receptor(s) and blocking fusion of viral and host membranes are likely to be key mechanisms of neutralization. Preventing cathepsin cleavage is another formal possibility but remains controversial [28], [29]. The single human MAb KZ52 did not protect rhesus macaques from lethal ZEBOV challenge given a higher dose of MAb (50 mg/kg, two times) [18] than that used in this NHP study (approximately 10–13 mg/kg, 3 times). Thus, the combination of two MAbs as done in this study seems to have improved treatment efficacy at lower antibody doses. Efficacy might be even higher with a cocktail of three or more MAbs, in particular if they target distinct epitopes and thus have independent mechanisms of action.

Previous studies on viral vector-based EBOV vaccine have suggested that the induction of cellular immune responses is also an important protective mechanism for EBOV infection [30], [31]. Since dysfunction of the immune system is critical for the pathogenesis of EHF in humans and NHPs [6], [8], [32], strategies need to be developed improving the immune functions (both humoral and cellular) disrupted during EHF. Thus, combined treatment with neutralizing MAbs and immune-modulating compounds should be evaluated in a future NHP study.

## Materials and Methods

### Challenge virus

ZEBOV (strain Kikwit) (kindly provided by the Centers for Disease Control and Prevention, Atlanta, Georgia, USA) was propagated in commonly used African green monkey kidney Vero E6 cells (kindly provided by Dr. R. Baric, University of North Carolina, Chapel Hill, NC, USA). The supernatants were cleared of cell debris, aliquoted, and stored in liquid nitrogen until used. All infectious work with ZEBOV was performed in the biosafety level 4 (BSL-4) laboratories at the Integrated Research Facility in the Rocky Mountain Laboratories (RML), Division of Intramural Research, National Institute of Allergy and Infectious Diseases (NIAID), National Institutes of Health (NIH), Hamilton, Montana, USA.

### Monoclonal antibodies

**Figure 5:**
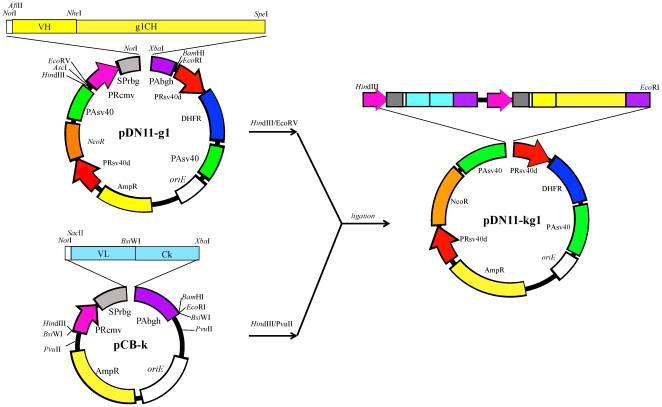
Scheme for the construction of pDN11-g1k. VH, heavy chain variable domain cDNA; VL, light chain variable domain cDNA; PAsv40, simian virus 40 terminator; PRcmv, cytomegalovirus promoter; g1CH, constant domain of human IgG1; Ck constant domain of human kappa chain; PRsv40d, enhancerless simian virus 40 promoter; NeoR, modified neomycin phosphotransferase gene; PAsv40, SV40 polyadenylation signal; PAbgh, bovine growth hormone gene terminator; SPrbg, rabbit beta-globin intron; AmpR, β-lactamase gene; DHFR, dihydrofolate reductase cDNA; oriE, replication origin of pBR322 plasmid.

Total RNA was extracted from mouse hybridoma cells producing mouse MAb ZGP133/3.16 or ZGP226/8.1, both of which were shown previously to neutralize ZEBOV [15], [19]. The variable heavy- and light-chain regions were amplified by RT-PCR with primers designed for these antibodies. The PCR products were subcloned into the pBR322-based plasmid, heavy- and light-chain (IgG1) construction vectors (pDN11-g1 and pCB-k, respectively), and the light chain cassette was transferred from pCB-k into the heavy-chain expression vector pDN11-g1 (Figure 5). The resulting plasmids (pDN11-kg1) expressing human-mouse chimeric MAbs ch133 and ch226 were designated DN11-ch133kg1 and DN11-ch226kg1, respectively. Stable cell lines expressing recombinant MAbs ch133 and ch226 were obtained by transfection of CHO DG44 cells (Invitrogen) with DN11-ch133kg1 and DN11-ch226kg1, respectively. Forty-eight hours after transfection, cells were seeded at a density of 1×10^3^ cells/0.1 ml/well onto 96-well microculture plates in IS-CHO CD medium (Irvine Scientific) containing G418 (400 µg/ml). Two weeks after transfection, proliferating clones were isolated and transferred to 24-well plates. Culture supernatants were collected and analyzed for production of each MAb (ch133 and ch226) by ELISA. For each MAb, the clone showing the highest expression level was propagated further. A chimeric MAb (ch61) specific for influenza virus hemagglutinin [strain A/Viet Nam/1194/2004 (H5N1)] was generated as a control MAb using the same methodology. MAb-expressing cell clones were maintained in IS-CHO CD medium and the recombinant MAbs ch133, ch226, and ch61 were purified from culture supernatants using rProtein A Sepharose Fast Flow (GE Healthcare) and Endospecy ES-50M (Seikagaku Biobusiness Corporation). MAb purity (98%<) and endotoxin levels (<1.0 EU/ml) were confirmed by performing SDS-PAGE and an EndoTrap red test (Profos AG), respectively.

### Neutralization assay

Vero E6 cells were seeded in a 48-well plate to generate a confluent monolayer on the day of infection. MAb dilutions were prepared in DMEM supplemented with 2% fetal bovine serum and 25 µl were incubated with 200 focus forming units of ZEBOV in a total volume of 50 µl. After 30 min at 37°C the media was removed from cells, the serum-virus mixture was added and incubated for 60 min at 37°C. Then the mixture was removed from the cells and 0.5 ml of a 1.2% carboxymethyl cellulose (CMC)/MEM (Life Technologies) solution was added per well. Following incubation for 4 days at 37°C the plates were fixed with 10% neutral buffered formalin and removed from BSL4 laboratories according to standard operating procedures. Subsequently, the cells were permeabilized and foci were stained with a rabbit anti-VP40 antibody (kindly provided by Dr. Y. Kawaoka, University of Wisconsin, Madison, WI) followed by a FITC-labeled secondary antibody (Sigma). Foci were counted using a fluorescent microscope (Carl Zeiss Microimaging LLC).

### Passive immunization and protection experiments

In the prophylactic treatment study, rhesus macaques (male adults, 4.0–5.2 kg) were given a mixture of MAb ch133 and ch226 (25 mg each/animal; total of 50 mg per animal) (n = 3) or ch61 (50 mg/animal) (n = 1) intravenously 24 hours prior (day −1) to challenge with an intramuscular injection of 10^3^ plaque-forming units of ZEBOV. The same amounts of antibodies were administered again using the same route 24 and 72 hours after challenge. Blood samples were collected throughout the study (on days −1, 0, 1, 3, 5, 8, 11, 16, 21, 27, and 31), and used to determine virus titers and antibody concentrations. Animals were monitored daily for clinical signs (fever, posture, respiration, feces/urine, food intake, recumbence, attitude, and skin turgor) using a previously published scoring sheet approved by the local Institutional Animal Care and Use Committee (IACUC) at NIAID, NIH [33].

### Hematology and serum biochemistry

The total white blood cell count, lymphocyte, platelet, reticulocyte and red blood cell counts, hemoglobin, hematocrit values, mean cell volume, mean corpuscular volume, and mean corpuscular hemoglobin concentrations were determined from EDTA blood with the HemaVet 950FS+ laser-based hematology analyzer (Drew Scientific). Plasma biochemistry was analyzed from heparin blood using the blood chemistry analyzer iSTAT1 (Abbott Point of Care). Urea nitrogen, glucose, chloride, sodium, potassium, hematocrit, hemoglobin, pH, PCO2, TCO2, base excess, and anion gap values were determined using the EC8+ Cartridge. Creatinine values were evaluated using Crea cartridges.

### Enzyme-linked immunosorbent assay (ELISA)

The filovirus GP-based ELISA was performed as described previously [34]. Briefly, ELISA plates (Nunc Maxisorp) were coated with purified soluble ZEBOV GP lacking transmembrane domain (100 ng/50 µl/well), followed by blocking with 3% skim milk (200 µl/well). Serial dilutions of NHP serum samples and purified antibodies (ch133, ch226, and ch61) of known concentrations were prepared, added to the ELISA plates and incubated for 1 hour at room temperature. Bound antibodies were visualized by adding a secondary peroxidase-conjugated goat anti-human IgG Fcγ fragment antibody (Jackson ImmunoResearch) and 3,3′,5,5′-tetramethylbenzidine (Sigma). The addition of 1 M phosphoric acid stopped the reaction and the optical density (OD) at 450 nm was measured. Antibody concentrations in the NHP serum samples were determined based on the OD values obtained for the standard curves from purified ch133, ch226, and ch61. For ch61, A/Viet Nam/1194/2004 (H5N1) virus particles treated with 0.5% Triton X-100 were used as the ELISA antigen. ZEBOV NP-specific antibodies in the serum samples (1∶10000 dilution) were detected performing ELISA using a recombinant NP antigen [35] and peroxidase-conjugated goat anti-monkey IgG γ chain antibody (Rockland).

### Virus detection

Total RNA was isolated from whole blood samples using the QIAmp viral Mini RNA kit (Qiagen). All quantitative real-time RT-PCRs were performed by employing the QIAquick 1-step Rotorgene kit (Qiagen) and ZEBOV-specific primers and probes based on the nucleoprotein sequence (NP; bp 2661–2721, GenBank accession number AF086833). We performed virus titration by TCID_50_ in Vero E6 cells from the blood and selected tissue samples. Briefly, 10-fold serial dilutions of the blood and tissue homogenates were prepared and used to infect Vero E6 cells. Cells were monitored for cytopathic effects (CPE) and the TCID_50_ was calculated for each sample employing the Reed and Muench method [36].

### Animal ethics statement

Healthy, adult rhesus macaques (*Macaca mulatta*) were handled in the Rocky Mountain Laboratories (RML) Animal BSL-2 and BSL-4 containment space. Research was conducted in compliance with the Animal Welfare Act and other federal statutes and regulations relating to animals and experiments involving animals, and adhered principles stated in the Guide for the Care and Use of Laboratory Animals, National Research Council, 1996. The facility where this research was conducted (RML) is fully accredited by the Association for the Assessment and Accreditation of Laboratory Animal Care International and has an approved OLAW Assurance #A4149-01. Research was conducted under a protocol approved by the IACUC. All steps were taken to ameliorate the welfare and to avoid the suffering of the animals in accordance with the “Weatherall report for the use of non-human primates" recommendations. Animals were housed in adjoining individual primate cages allowing social interactions, under controlled conditions of humidity, temperature and light (12-hour light/12-hour dark cycles). Food and water were available ad libitum. Animals were monitored (pre- and post-infection) and fed commercial monkey chow, treats and fruit twice daily by trained personnel. Environmental enrichment consisted of commercial toys. All procedures were conducted by trained personnel under the supervision of veterinarians and all invasive clinical procedures were performed while animals were anesthetized. Early endpoint criteria, as specified by the IACUC approved score parameters, were used to determine when animals should be humanely euthanized.
